# Parents’ User Experience Accessing and Using a Web-Based Map of COVID-19 Recommendations for Health Decision-Making: Qualitative Descriptive Study

**DOI:** 10.2196/53593

**Published:** 2024-03-20

**Authors:** Samantha Cyrkot, Lisa Hartling, Shannon D Scott, Sarah A Elliott

**Affiliations:** 1 Alberta Research Centre for Health Evidence Department of Pediatrics University of Alberta Edmonton, AB Canada; 2 Cochrane Child Health Department of Pediatrics University of Alberta Edmonton, AB Canada; 3 Faculty of Nursing University of Alberta Edmonton, AB Canada

**Keywords:** awareness, COVID-19, credibility, credible, descriptive, guidelines, health evidence, information behavior, information needs, information seeking, information-seeking behaviour, interface, internet, interview, knowledge mobilization, parent, parenting, public health, qualitative, recommendation, recommender, SARS-CoV-2, think-aloud activity, think-aloud, trust, trustworthy, usability, user experience, web design, website

## Abstract

**Background:**

The eCOVID19 Recommendations Map & Gateway to Contextualization (RecMap) website was developed to identify all COVID-19 guidelines, assess the credibility and trustworthiness of the guidelines, and make recommendations understandable to various stakeholder groups. To date, little has been done to understand and explore parents’ experiences when accessing and using the RecMap website for COVID-19 health decision-making.

**Objective:**

To explore (1) where parents look for COVID-19 health information and why, (2) parents’ user experience when accessing and using the RecMap website to make health decisions, and (3) what knowledge mobilization activities are needed to increase parents’ awareness, use, and engagement with the RecMap website.

**Methods:**

We conducted a qualitative descriptive study using semistructured interviews and a think-aloud activity with parents of children aged 18 years or younger living in Canada. Participants were asked to provide feedback on the RecMap website and to “think aloud” as they navigated the website to find relevant COVID-19 health recommendations. Demographic information was collected using a web-based questionnaire. A hybrid deductive and inductive thematic approach guided analysis and data synthesis.

**Results:**

A total of 21 participants (13/21, 62% mothers) were interviewed and participated in a think-aloud activity. The data were categorized into four sections, representative of key elements that deductively and inductively emerged from the data: (1) parent information seeking behaviors and preferences for COVID-19, (2) RecMap website usability, (3) perceived usefulness of the RecMap website, and (4) knowledge mobilization strategies to increase awareness, use, and engagement of the RecMap website. Parents primarily used the internet to find COVID-19 information and focused on sources that they determined to be credible, trustworthy, simple, and engaging. As the pandemic evolved, participants’ information-seeking behaviors changed, specifically their topics of interest and search frequency. Most parents were not aware of the RecMap website before this study but found satisfaction with its concept and layout and expressed intentions to use and share it with others. Parents experienced some barriers to using the RecMap website and suggested key areas for improvement to facilitate its usability and perceived usefulness. Recommendations included a more user-friendly home page for lay audiences (separate public-facing user interface), improving the search and filter options, quicker navigation, clearer titles, more family-friendly graphics, and improving mobile-friendly access. Several strategies to disseminate the RecMap website were also expressed, including a mix of traditional and nontraditional methods (handouts and social media) in credible and high-traffic locations that parents frequent often.

**Conclusions:**

Overall, parents liked the concept of the RecMap website but had some suggestions to improve its usability (language, navigation, and website interface). These findings can be used to improve the RecMap website for parents and offer insight for the development and dissemination of effective web-based health information tools and resources for the general public.

## Introduction

During the COVID-19 pandemic, digital information sharing and exchange exploded as disease information emerged and messaging around various restrictions, such as lockdowns and physical distancing, came into effect. While this generated a wealth of web-based COVID-19 information, it presented challenges for the public, including parents and families, when navigating and recognizing trustworthy health recommendations [1,2].

In response, the eCOVID19 Recommendations Map & Gateway to Contextualization (RecMap) website was developed [3]. The overall aim of the RecMap is to identify and collate all COVID-19 guidelines, assess the credibility and trustworthiness of the recommendations, and make the recommendations understandable to various stakeholder groups. The RecMap was developed by Cochrane Canada in collaboration with the World Health Organization’s (WHO) Collaborating Center for Infectious Diseases, Research Methods and Recommendations at McMaster University, the GRADE (Grading of Recommendations Assessment, Development and Evaluation) centers, the Norwegian Institute of Public Health, the Guidelines International Network, the National Institute of Health and Care Excellence (NICE), WHO and the Pan American Health Organization (PAHO), and many other organizations [3,4]. The living catalogue of COVID-19 guidelines is freely accessible, in English and French [5].

On the RecMap website, the recommendations, which are typically written for health care professionals, are currently being adapted into plain language products. These products have been developed as easy-to-understand summaries of health recommendations [6,7]. Our group’s previous findings suggest that parents prefer and better understand plain language versions compared to standard language recommendations [8].

It is critical that the evidence-based information available to parents is effectively communicated, understandable, and engaging for lay audiences. This is necessary to guide uptake and create actionable impact [9-11]. However, previous anecdotal data have suggested that parents are unaware of the RecMap website when seeking COVID-19–related health information or recommendations. There is also little information about the RecMap website’s usability from the perspective of parents. Usability and perceived usefulness are key criteria to consider when developing effective web-based health information tools [12-15].

To address this gap, we aimed to explore (1) where parents look for COVID-19 health information and why, (2) parents’ user experiences (including barriers and facilitators) when accessing and using the RecMap website to make health decisions, and (3) what knowledge mobilization activities are needed to increase parents’ awareness, use, and engagement with the RecMap website. The results of this study can be used to inform the enhancement and knowledge mobilization of a web-based COVID-19 tool for the general public, as well as the future development of web-based resources on other health topics.

## Methods

### Ethical Considerations

Approval was received from the University of Alberta Health Research Ethics Board and the McMaster Research Ethics Board (Pro00126429, Pro15646), and all participants gave informed consent before any data collection. After study completion, participants were compensated with a CAD $50 (a currency exchange rate of CAD $1=US $0.6518 is applicable) electronic gift card for their time.

### Overview, Sampling, and Recruitment

We conducted a qualitative-descriptive study [16]. Participants were eligible and self-identified for study enrollment if they were a parent, legal guardian, or grandparent of a child aged 18 years or younger, were aged 18 years or older themselves, lived in Canada, could read and speak English, had access to email and the internet through a computer, tablet, or smartphone. The study was advertised nationwide on the web between February and April 2023 through our collaborators (eg, the RecMap Team, Pediatric Parent Consultancy Network, and Pediatric Parent Advisory Group), and networks (eg, Cochrane Canada, Translating Emergency Knowledge for Kids, and Children’s Healthcare Canada) through email, as well as on social media through Instagram, Twitter, and Facebook. Purposive sampling (based upon parenting role and ethnicity) was used to gather an in-depth understanding of Canadian parents’ experiences with the RecMap website [17]. Results are reported following the Consolidated Criteria for Reporting Qualitative Research checklist [18].

### Sample Size

Sample size was shaped by data saturation, which was assessed concurrently throughout data collection and analysis to assess data comprehensiveness, variation, richness, and redundancy [19].

### Study Components

#### Semistructured Interview and Think-Aloud Activity

Participants were invited to attend a web-based, one-on-one, semistructured interview using Zoom video conferencing software. The interview guide was field tested and adapted over 2 nonrecorded interviews using in-house research staff and a parent volunteer (Multimedia Appendix 1). Interviews were conducted by SC (a woman), who is a research coordinator with a Master of Science and has previous experience conducting qualitative interviews. Consenting participants had no previous relationship with the coordinator and were informed of the study objectives at the beginning of the interview. There were no other individuals present during each interview besides the participant and the research coordinator. Interviews were audio-recorded, deidentified, and transcribed verbatim using a third-party transcription service (SimplyTranscription). Field notes were made throughout the interviews, including during the think-aloud activity, in which the parent was observed navigating the website and shared their screen so the interviewer could ascertain the steps and clicks they used to complete the different tasks. The first part of the interview focused on understanding where parents look for COVID-19 information and for what purpose. Participants were then asked to visit the RecMap website on their electronic device of choice (eg, computer, tablet, or smartphone). The second part of the interview asked participants to provide feedback about the RecMap website and to “think aloud” as they were asked to navigate the website to find relevant health recommendations for children. The think-aloud activity is based in cognitive and psychological research, where participants talk aloud while performing a task to verbalize their thoughts that come to mind [20-22]. Think-aloud interview methods have formerly been used in combination to explore usability and perceived usefulness [12]. Participants were also asked to share possible knowledge mobilization strategies to increase parents’ awareness, use, and engagement with the RecMap website. No participants withdrew from the study, no repeat interviews were conducted, and transcripts were not returned to the participants for corrections or to provide feedback on the findings.

#### Demographic Questionnaire

Participants were asked to complete a short web-based demographic questionnaire (eg, parenting role, age, education, ethnicity, and child’s age) after finishing the interviews. Quantitative data were collected and managed using REDCap (Research Electronic Data Capture), hosted at the University of Alberta [23,24].

### Data Analysis

#### Thematic Analysis

Thematic analysis was used to synthesize and identify common behaviors, processes, and preferences described in the semistructured interviews. Data management and analysis were facilitated using NVivo 14 Software (version 14.23.1; QSR International).

Data collection and analysis occurred iteratively, with data collection occurring until data redundancy was achieved. Interviews were coded by SC (the primary coder) and categorized to facilitate the development of themes. A hybrid deductive and inductive approach guided analysis in which data were categorized into 4 components. An established framework, the Technology Acceptance Model, was used to deductively organize the data, and additional codes were inductively gathered as they emerged from the data [12-15,25]. The analysis was guided by the following six main steps: (1) comparing the transcript with the recording and revising to ensure alignment (data cleaning); (2) reading transcripts and data familiarization; (3) generating initial codes (using a code manual and testing the reliability of codes through verification by a second reviewer [SAE]); (4) summarizing data and identifying initial themes guided by the model (deductive) and coding themes that extend beyond the model (inductive); (5) connecting the codes, identifying themes, and generating a “thematic map;” and (6) corroborating and legitimating coded themes. The coding system was refined throughout the iterative data collection and analysis stages, using a secondary coder (SAE) to code approximately 10% of the transcripts and compare them to maintain intrarater reliability [26]. Any discrepancies between the reviewers were discussed and resolved through consensus. The focused codes were further refined through collaboration between the 2 authors into themes and subthemes that identified common factors contributing to parental preference, usability, and perceived usefulness. All codes and transcripts were then re-examined to ensure the consistency and accuracy of the interpretation. Preliminary findings and interpretations were continuously reviewed and discussed among the research team. Other strategies for maintaining rigor were followed, such as detailed study logs and audit trails to ensure transparency [26].

#### Demographic Analysis

Demographic data were downloaded from REDCap into Microsoft Office Excel (2019; Microsoft Corp), and frequency distributions were used to describe the study sample.

## Results

### Sampling and Demographics

A total of 21 parents, mostly mothers (13/21, 62%) living in Canada, participated in interviews lasting between 30 and 60 minutes from February to April 2023. Participant demographics are presented in Table 1. Briefly, all parents reported a postsecondary education, with 57% (12/21) having a graduate or postgraduate degree. Parents identified most often as White (11/21, 52%) and South Asian (4/21, 19%). The RecMap website was accessed during the interviews by parents using a desktop or laptop computer (19/21, 90%), a tablet (1/21, 5%), and a smartphone (1/21, 5%).

**Table 1:** Participant demographics (N=21). A currency exchange rate of CAD $1=US $0.6518 is applicable.

Characteristics		Frequency, n (%)
**Parenting role**		
	Mother	13 (62)
	Father	8 (38)
**Age (years)**		
	31-40	13 (62)
	41-50	7 (33)
	≥51	1 (5)
**Relationship status**		
	Partnered (married or common law)	20 (95)
	Prefer not to answer	1 (5)
**Ethnicity**		
	Black	1 (5)
	South Asian	4 (19)
	East Asian	1 (5)
	Middle Eastern	1 (5)
	White	11 (52)
	Mixed Race	2 (10)
	Prefer not to answer	1 (5)
**Income (CAD)**		
	50,000-74,999	3 (14)
	75,000-99,999	1 (5)
	100,000-149,999	8 (38)
	≥150,000	8 (38)
	Prefer not to answer	1 (5)
**Education**		
	Postsecondary certificate or diploma	1 (5)
	Postsecondary degree	8 (38)
	Graduate degree	11 (52)
	Postgraduate degree	1 (5)
**Number of children**		
	1	9 (43)
	2	11 (52)
	3	1 (5)
**Children’s age range (years)**		
	1-5	12 (35)
	6-10	17 (50)
	≥11	5 (15)

No participants identified as being a legal guardian, aged 30 years or younger, with an income of <CAD $50,000 (US $37,100.25), an education below postsecondary, having ≥4 children, or their child being aged 1 year or younger. All participants identified as being partnered, except for 1 participant who selected “prefer not to answer.”

### Semistructured Interview and Think-Aloud Activity

The data were categorized into 4 sections, each representative of key elements that deductively and inductively emerged from the data. The first section addressed our first study objective of parent information-seeking behaviors and preferences for COVID-19. The second and third sections addressed our second objective, where we analyzed user experience based on 2 dimensions, namely, RecMap website usability and perceived usefulness of the RecMap website. The fourth section addressed our final objective of knowledge mobilization strategies to increase awareness, use, and engagement with the RecMap website. Illustrative quotes to support themes and subthemes are presented in Multimedia Appendix 2.

### Parent Information-Seeking Behaviors and Preferences for COVID-19

Relevant parent information-seeking behaviors and preferences about COVID-19 were grouped into 3 themes described in subsequent sections.

#### Parents Seek COVID-19 Information From a Variety of Sources

Parents reported seeking COVID-19 information from a variety of sources. The majority of parents used the internet (eg, web-based search engines, visiting health websites, and social media) as their primary source and reported being very comfortable using this modality to look for information. Secondary sources included the news, radio, and respected friends, family, and health care providers. Parents sought information applicable to their local jurisdiction (ie, province and community) to ensure familiarity with their current guidelines and mandates.

#### Parents Changed Their Information-Seeking Behaviors During the COVID-19 Pandemic

Parents reported a change in their information-seeking behaviors throughout the pandemic, particularly related to their topics of interest and information-seeking frequency. At the start of the pandemic, many parents reported seeking COVID-19 information on a daily or weekly basis, and their topics of interest included COVID-19 signs and symptoms, isolation requirements, and case numbers. At the time of conducting these interviews, parents reported seeking information less frequently (eg, on a monthly basis or less) and revealed looking for information on topics such as vaccines, masking, or travel guidelines when relevant to their situation and when needing to make an informed health decision for their family.

#### Parents Look for Specific Features and Elements When Seeking COVID-19 Information

The majority of parents described looking for COVID-19 information that was credible and trustworthy, which they characterized as being from a reputable source (eg, health authorities and the government). Parents also looked for information that was convenient to access (eg, mobile-friendly through a smartphone), relevant to their current environment (eg, email directly from their workplace or their child’s school), and aesthetically pleasing (eg, simple and engaging).

### RecMap Website Usability

The data from the think-aloud activity were grouped into the following three themes related to RecMap website usability: (1) RecMap website purpose and target audience, which explored parents’ overall thoughts about the usefulness of the website; (2) RecMap website presentation and navigation, which explored their preferences for layout, formatting, language, and how parents used the website; and (3) RecMap website functionality, which highlighted parents’ experiences with website features and overall usability.

### RecMap Website Purpose and Target Audience

The purpose of the RecMap website was well understood by most parents; however, the purpose of the RecMap website was not described anywhere on its home page. Parents wanted clearer messaging on the home page about what the website could offer families and the types of health recommendations available for children.

The target audience of the RecMap website was described as “for everyone” (ie, academics, health care providers, and the public), but again, parents felt that the home page lacked content directly addressing the public (or lay audiences), especially families. Some parents also questioned if the RecMap website was intended for a Canadian or global audience, as some content seemed specific to Canada (eg, McMaster University and Cochrane Canada logos on embedded videos and the website being only available in English and French), while other content felt geared toward a broader population (eg, recommendations presented based on European sources).

### RecMap Website Presentation and Navigation

The overall layout and format of the RecMap website were appealing to parents and were described as professional and simple. However, some parents did not readily see who the website was developed by, particularly within the first few moments of navigating the home page, and this led them to initially question its credibility. The color scheme of the website was said to be aesthetically pleasing, but some parents felt that an extra pop of color with more family-friendly graphics could better engage a lay audience. Parents also noted the navigation buttons were not always intuitively located, and they found some headings to be repetitive, with the font size not consistently proportionate between headings, subheadings, and body text.

The recommendations in the format of a list compared to a map on the RecMap website were preferred by most parents, as they felt that it was more familiar to them and easier to understand and navigate (Figures S1 and S2 in Multimedia Appendix 3). However, many parents commented that the list was lengthy and wondered how it was organized (eg, alphabetically or by date), as that impacted their ability to find relevant information quickly.

Parents felt that some terminology used on the website (eg, adolopment and conditional) was too complex and that many lay audiences would struggle to understand the terms “RecMap” and “plain language,” which were frequently used. Parents noted that although the navigation button titled “plain language recommendations” was well-situated on the home page, they cautioned that parents who are unfamiliar with this term may not know how to navigate and seek out these recommendations, which are tailored to lay audiences. Parents cautioned that it may lead to others exploring standard language recommendations first when they could benefit from plain language. Parents suggested refraining from titling these recommendations as “plain language” and generating an alternative title that is clearer and more suitable for lay audiences.

Parents thought the website was easy to navigate, especially the home page, but found that it required several clicks to reach a specific health recommendation. Parents also found that navigating the recommendations in map view was challenging as many were unfamiliar with this format and required a few moments to understand what the headings and numbers signified (Figure S2 in Multimedia Appendix 3).

Parents used a variety of navigation strategies when asked to find a recommendation specific to children. Most parents’ initial instinct was to use the search function. Others clicked on a preset option that was located on the home page (ie, either the map, list, or plain language recommendations button). Parents described their strategy rationale as being based on a combination of comfort levels (eg, using the search function), convenience (eg, quick), and taking into consideration the existing features and information available on the website. Parents who clicked on the “plain language recommendations” option from the start often felt less overwhelmed with information compared to those who initially browsed the standard language recommendations. Additionally, parents noticed that when using the search function on the home page, the results generated standard language recommendations, but parents felt that results for plain language recommendations should be prioritized for lay audiences.

### RecMap Website Functionality

Parents found a variety of features to be useful and improve their user experience and overall website usability. The RecMap website’s search feature was emphasized by parents as appealing because it was well-located and gave the freedom to search for any topic of interest. However, challenges were experienced when the use of basic search terms led to unsuccessful findings (eg, irrelevant or no recommended results), and parents had to adjust their terms multiple times or try a new navigation strategy to obtain relevant results.

The filter option on the list of recommendations was frequently used by parents. This feature was reported as practical, but with some complex and irrelevant categories for a lay audience (eg, adolopment and grading approach). Based on the available categories, parents thought that age group (eg, infant, child, or youth), world region, source, and year of publication were most relevant and suggested adding age range. They also felt that these categories should be rearranged and listed at the top for easy access.

Parents were divided on whether they liked the feature of a new tab opening up (within their web browser) for each specific recommendation that they clicked on and explored. Some parents enjoyed this feature because they could easily navigate between tabs to different recommendations, but some did not notice the new tab, which made it challenging for them to return to the home page.

Parents noted a few undesirable features and some technical barriers as they explored the website. Many parents found that the mailing list pop-up (eg, subscribe to receive information about upcoming activities) was distracting and appeared too soon before they had a chance to explore the full website. Some parents also experienced extended loading times (eg, when trying to access the recommendations in map view), which impacted the overall usability and user experience of the website.

### Perceived Usefulness of the RecMap Website

Regarding perceived usefulness, two themes emerged as follows: (1) intentions to use the RecMap website, which looked at parents’ thoughts about its usefulness and their desire to share and discuss it with others and seek supportive information; and (2) parents’ awareness and expectations of the RecMap website, which explored whether parents were familiar with and had previously accessed the website and if it met their needs and expectations.

### Intentions to use the RecMap Website

After completing the think-aloud activity, most parents discussed their intentions to use and share the website with others (eg, friends and family). Parents who were hesitant to use or share it suggested that the home page needed to be more user-friendly (for a lay audience) with clearer titles, quicker navigation, and improved search and filter options to seek out relevant plain language recommendations in a timely manner.

Parents felt that if needed, they would discuss the RecMap website with their health care provider or seek out supplementary information (eg, other web-based sources) to enhance their understanding of what was available on the website in order to make an informed health decision for their child.

### Awareness and Expectations of the RecMap Website

Very few parents reported being aware of or previously accessing the RecMap website before this study. Parents suggested that their unawareness may be due to limited promotion, a lack of health care referral, or that the RecMap website did not appear in their search engine results for COVID-19.

The overall concept of the RecMap website was liked by most parents, and they perceived this tool to be useful. Parents highlighted that it gives users an opportunity to search for COVID-19 information from a credible source, and some positively compared it to a large COVID-19 web-based search engine.

Some parents expressed that parts of the RecMap website were not what they expected and wanted to see more information tailored for parents and lay audiences. This included addressing COVID-19 topics related to possible child COVID-19 complications and symptom management, which parents felt were not clearly addressed. Additionally, most parents did not expect that when navigating the website, they would have the option to see recommendations in both standard and plain language. This had taken many parents a few moments to realize, particularly those who were unfamiliar with the term “plain language.” They also wanted more clarification about what the recommendation map was compared to the recommendation list and if there was a difference between them beyond format.

### Knowledge Mobilization Strategies

Knowledge mobilization strategies were grouped into two themes: (1) recommendations to tailor the RecMap website for parents, which highlighted suggestions to make the website more user-friendly and accessible for parents; and (2) approaches to dissemination, which explored where and how to share information about the RecMap website within the parent community to increase awareness, use, and engagement.

### Recommendations to Tailor the RecMap Website for Parents

To better tailor the website for parents, it was often suggested to develop a separate user interface within the website to split the content into lay or public and health care professional or academic. This would facilitate relocating the standard language recommendations, which most parents find challenging to understand. This would also enable more tailored features, where some parents suggested a “frequently asked questions” section to dispel myths or an ability to insert anonymous data about your child (eg, age and vaccine status) into the RecMap website to generate relevant recommendations. A “bookmark” or “share” option to save recommendations was also suggested by parents, as they described having busy schedules and wanting quick and easy-to-find information.

Parents felt that the accessibility of the website was another key element to highlight. A mobile-friendly website and mobile apps were mentioned by many parents, who reported that they often use their smartphones as a convenient tool to look for information. Even though the RecMap is currently accessible through a smartphone, the map of recommendations is inaccessible. Parents also felt that being mindful of contrasting colors on the RecMap website was important for the accessibility of those with various visual abilities.

### Approaches to Dissemination

Parents shared a variety of dissemination strategies to increase awareness of the RecMap website within the parent community. Parents felt that sharing information in convenient and high-traffic locations where parents frequent often (eg, social media, such as Facebook, Instagram, Tik Tok, and Twitter; health and immunization clinics; schools; parent groups; at extracurricular activities or events such as soccer games) would increase parents’ awareness and thus website use. Other strategies included collaborating with credible and trusted organizations and individuals (eg, health authorities, health care providers, and schools) to share information through their networks in person or through a web-based presence.

Parents suggested that approaches to increasing awareness of the RecMap website should not be limited to just one but rather should consist of multiple captivating methods to attract and engage parents with different preferences and means to access information. Methods suggested included QR codes, handouts, posters, social media posts, advertisements and sponsors (eg, on the radio, podcasts, in parenting magazines, school newsletters), media interviews (eg, on the news and radio), and word of mouth from trusted individuals (eg, health care providers).

## Discussion

### Principal Findings

The aim of this study was to explore parental COVID-19 information-seeking behaviors and preferences, along with their experience when accessing and using the RecMap website. Parents also shared several knowledge mobilization strategies to facilitate parents’ awareness, use, and engagement of the RecMap website.

### Information-Seeking Behaviors and Preferences

This study identified that most parents report using the internet as a primary source to look for COVID-19 health information. This is not surprising given that recent reports document that approximately 70% of Canadians and Americans use the web to search for health information [27,28]. There is also now an array of COVID-19–specific information on the web that has become easily and conveniently accessible to the public [2]. Of note, parents reported a change in their information-seeking behaviors and preferences since the pandemic started, with parents looking for information less frequently as the pandemic evolved. This is important to consider when planning future knowledge mobilization strategies aimed at parents and the general public.

### Barriers to Using the RecMap Website

When parents initially accessed the RecMap website, most struggled to identify who developed it; thus, many parents questioned its credibility. Web-based tools that are immediately recognizable (eg, well-branded and clear messaging on the home page) may help increase credibility and should be considered for future knowledge mobilization efforts among the general public and lay audiences [29].

Additionally, there was some content and language within the RecMap website that was deemed too advanced or complex by participants and thus a barrier, such as recommendations that were written in standard language. However, some participants expressed benefits to having access to standard language and suggested a public-facing user interface to separate laypersons and the public from health care professionals or academic content. Similar interface designs have been used on other health-related information websites across Canada, including Alberta Health Service and Ontario Health [30,31]. This format (lay-friendly material upfront with the ability to access standard language content) will also provide more opportunities to customize the RecMap website for this population and address additional suggestions mentioned by participants (eg, messaging that is focused on what the RecMap website can offer the general public).

Parents also experienced technical barriers with the loading time of the recommendations in the map view. A study on web usability testing in parents found that technological difficulties were a key barrier to successfully using a website and its features [32]. Load time is also an important web performance metric that can directly impact user engagement. Websites that take longer than 3 seconds to load have reported that almost 55% of users leave the website [33]. In fact, some of our participants mentioned that they would potentially exit a website and find a new one if the loading time was extensively long. Other minor barriers noted in this study were shortcomings in the visual design (eg, color and graphics), unclear titles, and challenges with returning to the home page. Similar barriers have been reported in previous research that explored website user experience and usability testing [12,34], highlighting the need for end user input into the design at an early stage.

### Facilitators for Using the RecMap Website

While parents liked the concept and felt the RecMap website could be useful to them, they described several facilitators to optimize use. The concept of the RecMap has previously been used and implemented by the World Health Organization to house living guidelines and recommendations on tuberculosis prevention and care [35]. At this time, usability testing has not been formally completed with parents or the general public. However, the RecMap’s digitized dissemination format has been compared for usability to a conventional tuberculosis website containing guideline recommendations, and the RecMap was found to be more accessible, improve understanding, and enable decision-making [36].

Other facilitators for parents using the RecMap website included a professional and simple presentation, the availability of an internal search engine, and a home page that was easy to navigate. These suggested elements are in line with standards that have been identified for the development of digital-based health information resources. In particular, when information is aesthetically pleasing, easy to find and navigate, this can contribute to increased user trust, readability, and facilitation of understanding [37]. Other research has found that simple aesthetics [29], clearly described headings and labels, information that is logically organized [38], limited technological barriers [32], and being usable in stressful situations [29] are equally preferred by parents. The use of tailored features to meet the needs and preferences of parents was also suggested to improve website usability and quality [32]. This was recommended by our participants in the form of a frequently asked questions section or the ability to bookmark recommendations for easy access.

### Knowledge Mobilization Strategies

In 2018, it was reported that almost 90% of Canadian internet users aged 15 years and older have a smartphone and that almost 50% check their smartphone at least every 30 minutes [39]. Many parents in our study reported that they typically use their smartphones to look for and access health-related information, however, the initial version of the RecMap was not specifically designed to be smartphone compatible. It is clearly important that health-based websites are mobile-friendly and compatible on devices with smaller screens to optimize viewing and ease of navigation. Better accessibility may also support user experience, which can lead to better user engagement and increase usage.

Mobile apps are another avenue to explore for the RecMap, as there is a rise in parents using apps to support their parenting [40]. This modality is versatile and can encompass multimedia elements (eg, graphics and videos) to engage users and push notifications to alert users of new information or updates on the application. This is an interesting feature to consider given that the RecMap contains living recommendations that are frequently updated or changed. It is equally important for parents that mobile apps are of high quality, relevant, visually appealing, interactive, and seamless (ie, easy to navigate, do not freeze, or crash); otherwise, there is a risk that flawed apps will be deleted by end users within minutes after being downloaded [40].

It has been previously reported that dissemination strategies appropriate for parents include targeting credible sources such as community centers, public health institutions, schools, physicians, and government agencies [41]. This is comparable to our findings, where parents suggested targeting schools, health clinics, and collaborating with credible and trusted organizations and individuals to increase the RecMap website’s awareness. Furthermore, collaborating with reputable messengers (eg, physicians) can be an effective strategy. In fact, it is suggested that 70% of patients would like their physician to recommend web-based health information for them to review [42]. Similar to our findings, it has also been suggested that using a combination of dissemination methods can be most effective. This includes a multiplatform approach of digital (eg, social media and websites), traditional (eg, brochures and radio), and unique means (eg, community “parent nights”) [41].

### Strengths and Limitations

This work has important implications for knowledge mobilization strategies to enhance and contextualize products to meet diverse end users’ needs as we move beyond the pandemic and for other public health issues, including regular seasonal outbreaks and future epidemic approaches.

A strength of this study is the use of multiple methods. The think-aloud activity allowed us to collect direct data on user experience and parent thoughts while using the RecMap website, and the interviews provided us with an opportunity to clarify initial thoughts and ask further questions. Nevertheless, the majority of participants self-identified as White and of higher socioeconomic status, which limited the generalizability of study findings to a wider audience and was not representative of Canada’s ethnically diverse population. However, we were able to recruit participants who identified as both mothers (13/21, 62%) and fathers (8/21, 38%), which enabled us to gather differing perspectives in terms of parenting roles. Future studies may benefit from developing a recruitment strategy that targets those with different parenting roles, visible minority groups, those of lower socioeconomic status, and those living in geographically diverse locations (eg, rural). In doing so, a better understanding of the diverse needs of parents across Canada could be examined, and strategies (digital and nondigital) to support knowledge mobilization around COVID-19 in various contexts could be developed. It is also worth noting that all participants lived in Canada and had access to a similar health care setting.

### Directions for Future Research

Future work related to the RecMap website should focus on using an integrated knowledge translation model where end users (eg, parents) codevelop and implement a plan to increase the awareness, use, and engagement of the website among the general public and lay audiences. Our group will also work to improve the usability of the RecMap website and implement the changes based on our findings related to parents’ user experiences. This may also include further work on mobile-friendly access (eg, smartphones and mobile apps). These steps will enhance the existing RecMap website and ensure that it is user-friendly and meets the needs of parents and the general public. Future projects similar to RecMap that seek to share health recommendations should involve various end users (eg, parents, health care providers, and the general public) during the conceptual phase of web-based tool development to better tailor the final product toward their specific goals and needs.

As public health approaches to COVID-19 evolve and the focus shifts to post–COVID-19 conditions, researchers developing knowledge mobilization strategies can use these findings to inform decisions for implementing knowledge. While the RecMap was not developed specifically to meet parents’ information needs during the pandemic, it provides a model for how rapidly changing evidence can be synthesized and presented to support decision-making. However, rapidly changing contextual factors may affect the potential use, relevance, and positioning of the RecMap to support knowledge needs around COVID-19 in the future. The waning of attention to COVID-19 guidelines internationally and jurisdictional changes to COVID-19 management question the advantages of resources such as the RecMap post pandemic. Future research should explore sustainable models of knowledge mobilization to support parental information-seeking behaviors. The pandemic highlighted how families engage with web-based health information and how changes in knowledge needs and information-seeking behaviors need to be considered.

### Conclusions

This study reports on the COVID-19 information-seeking behaviors and preferences of parents, the RecMap website user experience (including barriers and facilitators), and potential knowledge mobilization strategies. The concept of the RecMap website was appealing to parents and has the potential to become a user-friendly web-based tool for parents. However, understanding how, when, and why parents search for COVID-19 information, as well as what aspects of the user experience can be improved, has provided meaningful insight and recommendations to improve the usability and perceived usefulness of the RecMap website. Additionally, appropriately tailored knowledge mobilization strategies targeted at parents will increase their awareness, use, and engagement of the RecMap website. This data will contribute to the enhancement of the COVID-19 RecMap website for parents and can inform the development of effective web-based tools for the general public about other health topics.

## Appendix

Multimedia Appendix 1 Interview guide questions and think-aloud activity processes.

Multimedia Appendix 2 Illustrative quotes to support the themes and subthemes.

Multimedia Appendix 3 The a) list and b) map view of the COVID-19 recommendations available on the RecMap website.

## Abbreviations

GRADE: Grading of Recommendations Assessment, Development and Evaluation
NICE: National Institute of Health and Care Excellence
PAHO: Pan American Health Organization
RecMap: Recommendations Map & Gateway to Contextualization
REDCap: Research Electronic Data Capture
WHO: World Health Organization

## References

[1] Mian A, Khan S (2020). Coronavirus: the spread of misinformation. BMC Med.

[2] Tangcharoensathien V, Calleja N, Nguyen T, Purnat T, D'Agostino M, Garcia-Saiso S, Landry M, Rashidian A, Hamilton C, AbdAllah A, Ghiga I, Hill A, Hougendobler D, van Andel J, Nunn M, Brooks I, Sacco PL, De Domenico M, Mai P, Gruzd A, Alaphilippe A, Briand S (2020). Framework for managing the COVID-19 infodemic: methods and results of an online, crowdsourced WHO technical consultation. J Med Internet Res.

[3] Lotfi T, Stevens A, Akl EA, Falavigna M, Kredo T, Mathew JL, Schünemann HJ (2021). Getting trustworthy guidelines into the hands of decision-makers and supporting their consideration of contextual factors for implementation globally: recommendation mapping of COVID-19 guidelines. J Clin Epidemiol.

[4] About us. eCOVID RecMap.

[5] eCOVID RecMap.

[6] Santesso N, Wiercioch W, Barbara AM, Dietl H, Schünemann HJ (2022). Focus groups and interviews with the public led to the development of a template for a GRADE Plain Language Recommendation (PLR). J Clin Epidemiol.

[7] Pottie K, Smith M, Matthews M, Santesso N, Magwood O, Kredo T, Scott S, Bayliss K, Saad A, Haridas R, Detambel N, Motilall A, Tan Y, Steinberg S, Litynska J, Dietl B, Ioiri A, Reveiz L, Welch V, Klugar M, Mbuagbaw L, Rojas MX, Florez ID, Lotfi T, Qaseem A, Mathew JL, Akl EA, Tugwell P, Schünemann HJ (2022). A multistakeholder development process to prioritize and translate COVID-19 health recommendations for patients, caregivers and the public. A case study of the COVID-19 recommendation map. J Clin Epidemiol.

[8] Elliott SA, Scott SD, Charide R, Patterson-Stallwood L, Sayfi S, Motilall A, Baba A, Lotfi T, Suvada J, Klugar M, Kredo T, Mathew JL, Richards DP, Butcher NJ, Offringa M, Pottie K, Schünemann HJ, Hartling L (2023). A multimethods randomized trial found that plain language versions improved parents' understanding of health recommendations. J Clin Epidemiol.

[9] Hyland-Wood B, Gardner J, Leask J, Ecker UKH (2021). Toward effective government communication strategies in the era of COVID-19. Humanit Soc Sci Commun.

[10] Holroyd TA, Oloko OK, Salmon DA, Omer SB, Limaye RJ (2020). Communicating recommendations in public health emergencies: the role of public health authorities. Health Secur.

[11] Risk communication and community engagement: a compendium of case studies in times of COVID-19. World Health Organization.

[12] Bolle S, Romijn G, Smets EMA, Loos EF, Kunneman M, van Weert JCM (2016). Older cancer patients' user experiences with web-based health information tools: a think-aloud study. J Med Internet Res.

[13] Davis FD (1986). A technology acceptance model for empirically testing new end-user information systems: theory and results. Sloan School of Management, Massachusetts Institute of Technology.

[14] Lee Y, Kozar KA, Larsen KRT (2003). The technology acceptance model: past, present, and future. Commun Assoc Inf Syst.

[15] Holden RJ, Karsh BT (2010). The technology acceptance model: its past and its future in health care. J Biomed Inform.

[16] Sandelowski M (2004). Using qualitative research. Qual Health Res.

[17] Palinkas LA, Horwitz SM, Green CA, Wisdom JP, Duan N, Hoagwood K (2015). Purposeful sampling for qualitative data collection and analysis in mixed method implementation research. Adm Policy Ment Health.

[18] Tong A, Sainsbury P, Craig J (2007). Consolidated criteria for reporting qualitative research (COREQ): a 32-item checklist for interviews and focus groups. Int J Qual Health Care.

[19] Guest G, Namey E, Chen M (2020). A simple method to assess and report thematic saturation in qualitative research. PLoS One.

[20] Charters E (2003). The use of think-aloud methods in qualitative research an introduction to think-aloud methods. Brock Educ J.

[21] Ericsson KA, Simon HA (1980). Verbal reports as data. Psychol Rev.

[22] Jaspers MWM, Steen T, van den Bos C, Geenen M (2004). The think aloud method: a guide to user interface design. Int J Med Inform.

[23] Harris PA, Taylor R, Minor BL, Elliott V, Fernandez M, O'Neal L, McLeod L, Delacqua G, Delacqua F, Kirby J, Duda SN (2019). The REDCap consortium: building an international community of software platform partners. J Biomed Inform.

[24] Harris PA, Taylor R, Thielke R, Payne J, Gonzalez N, Conde JG (2009). Research Electronic Data Capture (REDCap)--a metadata-driven methodology and workflow process for providing translational research informatics support. J Biomed Inform.

[25] Fereday J, Muir-Cochrane E (2006). Demonstrating rigor using thematic analysis: a hybrid approach of inductive and deductive coding and theme development. Int J Qual Methods.

[26] Morse JM (2015). Critical analysis of strategies for determining rigor in qualitative inquiry. Qual Health Res.

[27] (2021). Table 22-10-0137-01. Selected online activities by gender, age group and highest certificate, diploma or degree completed. Statistics Canada.

[28] Rutten LJF, Blake KD, Greenberg-Worisek AJ, Allen SV, Moser RP, Hesse BW (2019). Online health information seeking among US adults: measuring progress toward a healthy people 2020 objective. Public Health Rep.

[29] Anzinger H, Elliott SA, Hartling L (2020). Comparative usability analysis and parental preferences of three web-based knowledge translation tools: multimethod study. J Med Internet Res.

[30] (2023). Alberta Health Services.

[31] (2023). Ontario Health.

[32] Cook RS, Rule S, Mariger H (2016). Parents' evaluation of the usability of a web site on recommended practices. Top Early Child Spec Educ.

[33] Agrawal G, Kumar D, Singh M (2021). Assessing the usability, accessibility, and mobile readiness of e-government websites: a case study in india. Univ Access Inf Soc.

[34] Starling R, Nodulman JA, Kong AS, Wheeler CM, Buller DB, Woodall WG (2015). Usability testing of an HPV information website for parents and adolescents. Online J Commun Media Technol.

[35] Hajizadeh A, Lotfi T, Falzon D, Mertz D, Nieuwlaat R, Gebreselassie N, Jaramillo E, Korobitsyn A, Zignol M, Mirzayev F, Ismail N, Brozek J, Loeb M, Piggott T, Darzi A, Wang Q, Mahmood AS, Saroey P, Matthews M, Schünemann F, Dietl B, Nowak A, Kulesza K, Muti-Schünemann GEU, Bognanni A, Charide R, Akl EA, Kasaeva T, Schünemann HJ (2021). Recommendation mapping of the World Health Organization's guidelines on tuberculosis: a new approach to digitizing and presenting recommendations. J Clin Epidemiol.

[36] Matthews M, Lotfi T, Santesso N, Loeb M, Mertz D, Chagla Z, Hajizadeh A, Piggott T, Dietl B, Schünemann HJ (2022). Comparing the usability of the World Health Organization's conventional tuberculosis guidelines to the eTB recommendations map: a two-arm superiority randomised controlled trial. PLOS Glob Public Health.

[37] Abdel-Wahab N, Rai D, Siddhanamatha H, Dodeja A, Suarez-Almazor ME, Lopez-Olivo MA (2019). A comprehensive scoping review to identify standards for the development of health information resources on the internet. PLoS One.

[38] Kokil U, Scott S (2017). Usability testing of a school website using qualitative approach. Proceedings of the 12th International Joint Conference on Computer Vision, Imaging and Computer Graphics Theory and Applications (VISIGRAPP 2017), Vol 2.

[39] (2021). Table 22-10-0115-01. Smartphone use and smartphone habits by gender and age group, inactive. Statistics Canada.

[40] Virani A, Duffett-Leger L, Letourneau N (2021). Parents’ use of mobile applications in the first year of parenthood: a narrative review of the literature. Health Technol.

[41] Larocca V, Arbour-Nicitopoulos KP, Tomasone JR, Latimer-Cheung AE, Bassett-Gunter RL (2021). Developing and disseminating physical activity messages targeting parents: a systematic scoping review. Int J Environ Res Public Health.

[42] Oktay LA, Abuelgasim E, Abdelwahed A, Houbby N, Lampridou S, Normahani P, Peters N, Jaffer U (2021). Factors affecting engagement in web-based health care patient information: narrative review of the literature. J Med Internet Res.

