# Supplementary material for: Parents’ User Experience Accessing and Using a Web-Based Map of COVID-19 Recommendations for Health Decision-Making: Qualitative Descriptive Study
Source: JMIR Form Res. 2024 Mar 20;8:e53593. doi: 10.2196/53593 (PMC10956570; doi:10.2196/53593)
Supplement: Multimedia Appendix 1 [file formative_v8i1e53593_app1.pdf]

	Main Questions	Probes	Notes
**1**	Where do you usually search for COVID-19 health recommendations?	• Why do you go to the places mentioned?<br>• How often do you look for COVID-19 information?	
**2**	What do you look for in a website for it to be a website that you would go to and use?	• What elements do you look for?<br>○ Usable, useful, desirable, valuable, findable, credible, accessible, trustworthy<br>□ What does this mean to you?	
**3**	What would prevent you from going to and using a website?	• What format or language do you find unhelpful?	
**4**	Have you ever accessed the RecMap website before?	• Why? / Why not?	
**5**	What are your initial thoughts about this website?	• Do you know what this website is about?<br>• Who do you think this website is for?<br>• What do you think about the headers, descriptions and other features on the website? Are they easy to use? What makes it easy to use? Are they clear? What makes it clear?<br>• What do you think about the language used? Is it easy to understand?<br>• What do you like the most? Why?<br>• What do you like the least? Why?	*Prior to asking question 5, ask the participant to pull up the RecMap website on their web browser. If needed, probes can be asked after the think-aloud activity has been completed for this question.*
**6**	Can you try and find a COVID-19 related health recommendation for children?	• What did you do?<br>• How was your experience to find the recommendation?<br>• Is it clear what a 'recommendation good practice' statement versus 'additional guidance' is?<br>• What do you like about the layout? Why?<br>• What do you dislike about the layout? Why?	*Ask the participant to return back to the home page before asking question 6. If needed, probes can be asked after the think-aloud activity has been completed for this question.*
**7**	Can you try and find a COVID-19 related plain language recommendation for children?	• What did you do?<br>• How was your experience to find the recommendation?<br>• What do you like about the layout? Why?<br>• What do you dislike about the layout? Why?<br>• What do you think about the language used?	*Ask the participant to return back to the home page before asking question 7. If needed, probes can be asked after the think-aloud activity has been completed for this question.*

**8**	If you were using this site, which category would you click on first and why?<br><br>• Recommendations OR<br>• Plain language recommendations	• Is the difference between the two categories clearly explained on the website?<br>• If you click on 'recommendations', would you choose 'map' or 'list' first and why?<br>• Would you use the search option?	*Ask the participant to return back to the home page before asking question 8.*
**9**	Do you think this site is user-friendly for parents?	• Why?/ Why not?<br>• What do you think is something preventing parents from using the website?<br>• Now that you know about this website, would you use this website?<br>• If friends (other parents) were looking for COVID-19 recommendations, would you recommend it to them?<br>• Would you discuss the information that you found on this website with your doctor?<br>• How could you see parents using this website and the information available?<br>• Is this website what you expected it to be?	
**10**	What parts of this website could be improved?	• What about it did you not find useful?	
**11**	How do you think we could improve parents' awareness about the RecMap website?	• Where would you like to see this website shared?<br>• How would you like to see this website shared?	
**12**	Is there anything else you want us to know?	• Any additional comments or questions that we have not covered?	

COVID-19, Coronavirus Disease 2019; RecMap, Recommendations Map & Gateway to Contextualization.
