# Supplementary material for: Parents’ User Experience Accessing and Using a Web-Based Map of COVID-19 Recommendations for Health Decision-Making: Qualitative Descriptive Study
Source: JMIR Form Res. 2024 Mar 20;8:e53593. doi: 10.2196/53593 (PMC10956570; doi:10.2196/53593)
Supplement: Multimedia Appendix 2 [file formative_v8i1e53593_app2.pdf]

Element	Themes	Subthemes	Illustrative Quotes
**1) Parent information-seeking behaviours and preferences for COVID-19**	i. Parents seek COVID-19 information from a variety of sources	Parents use the internet as a source to seek for COVID-19 information	*'Government websites largely would be my first step…and probably Twitter if I had to pick a platform in social.'* P13
		Parents use other sources to seek for COVID-19 information	*'…sometimes I would be watching the national news when we first had COVID and [the Chief Medical Officer of Health], I listened to [them] for months. I would say for the daily report when [they were] doing that regularly.' P2*
	ii. Parents changed their information-seeking behaviours during COVID-19	Parents changed their topics of interest	*'I did a lot of searching on symptoms…at one time it was the whole kitchen sink was a symptom for COVID… And I also looked for information about prevalence in the community and number of hospitalizations, number of deaths, that sort of thing… Probably the only reason I would look for it now would be to understand vaccination schedules for my children.' P23*
		Parents changed their frequency of searching	*'Much less now. In the height of the pandemic, I would say I was looking quite frequently cause as I mentioned earlier, my youngest daughter has special needs and we're very unsure of how this would affect her.... But I think as we are now, I would probably look at it, I don't even know if I would once a month at this stage.' P13*<br>*'I think as things have evolved and will obviously continue to evolve -- if there was something I wanted to know a little bit more about. For example, all of a sudden, we're having flu shots. Well, if I just had my 4th booster or my children most importantly did, how long should I wait in between that?... I don't think this is something at this time in the pandemic or endemic that I would be able to want to look at this daily. But I mean, there were times throughout that I certainly would when things were changing more rapidly in my opinion.' P1*
	iii. Parents look for specific features and elements when seeking COVID-19 information	Parents look for information that is credible and trustworthy	*'I'll pop it into Google and see what comes up. Obviously knowing that not everything out there is reputable so just kind of weeding through and kind of searching, you know, taking into consideration different resources.' P16*<br>*'For me, it needs to be credible for sure. I need to know the source and I need to know that it's a legitimate source. And then my preference is that it's easy to use but certainly some of the healthcare ones are not easy to use but I still [use] it because I thought it was credible.' P2*

		Parents look for information that is convenient to access and relevant to their current environment	*'I'd say most of the time, I'm probably looking these things up on my phone so it would need to be compatible with that format.' P9*<br>*'I don't need to see all of the different recommendations from all over the world, what probably is most important are the ones that are right around where I am.' P6*
		Parents look for content that is aesthetically pleasing	*'I think it needs to be clean, not too content heavy because that can get overwhelming, especially something with COVID where there is a lot of information. I think the information needs to be well organized and not overwhelming.' P13*<br>*'So, it might seem childish, but when you're looking for kid's information, a website that has like a pop up of colour…you know, animated stuff that attracts a little bit…that is clear and the font size [is good] and everything.' P11*
**2) RecMap website usability**	i. RecMap website purpose and target audience	Parents understood the purpose of the RecMap website but wanted clearer messaging	*'I would say COVID-19 recommendations. I would say advice and recommendations for COVID-19. I would say probably general recommendations.' P7*<br>*'I think that when you come to it, you're not quite sure what you're going to use it for, who it's by, just having more information as soon as you open the website.' P12*<br>*'I see you have a list of recommendations. So, maybe explaining what kind of recommendations you are providing… a bit more information about what kind of things are covered, what kind of topics.' P21*
		Parents thought the target audience was 'everyone' but felt a lack of content directed at the public	*'I would think, kind of anybody affected by COVID, to be honest, I don't think it looks tailored to, you know, just parents or just adults or anything like that.' P13*<br>*'I wouldn't feel this was inviting to a parent. I mean to me maybe somebody that has like that scientific background… I think that's kind of where a little more imaging might be a bit more welcoming, right. It looks business to me. It looks a little more formal versus something that would be targeted at a family, right, parents.' P16*
	ii. RecMap website presentation and navigation	Parents liked the overall layout but some elements could be improved	*'I mean I think it's kind of like a basic layout, but I like it at the same time. Like it's not super flashy or anything like that.' P7*<br>*'…more imaging maybe just to make it a little more engaging I think.' P16*
		Parents preferred a list compared to a map format	*'I don't like this [map] version, to be honest. I find it very tricky to nail down what I want because you're going from a search term or something in general that you're looking for to nailing it down to two terms, basically and finding that correlation. So yeah, I do find that a little bit hard.' Yes [I prefer the list format].' P13*

			'I think I prefer having it laid out in the plain language [list] way so I can see it's clearly defined. This [map], I find very jumbled…I would be like, yeah, I'm not going to really mess around with this layout unless I really knew how to use it.' P8
		Parents found some language on the RecMap website to be too complex	*'I'm clicking on plain language recommendation. I didn't know if that was -- perhaps the website was translated into different languages, but it doesn't look like that is what is there.' P1*<br><br>*'…this [standard language version] absolutely looks like I've stumbled into medical journal stuff. Like there's no language at all that speaks to me as a parent…you know thinking about my husband, my sister, my friends, no one is gonna find this website useful. It's just like overwhelming.' P9*<br><br>*'And then there's a button there that say request for adolopment. I have no idea what that word even means.' P5*
		Parents found the overall RecMap website easy to navigate but experienced some barriers. They also used various navigation strategies to find information.	*'No. It's not too difficult to navigate, I think, because it's listed all the different topics on the side. And then it's got -- on the other side. I think it's quite easy to navigate. If you're looking for something specific, you can generally find it, I think.' P21*<br><br>*'I had to click an awful lot to get to this plain language thing. So, that was already one barrier.' P5*<br><br>*'We've got a recommendations map. List of recommendations. Plain language recommendations. So, that looks like I got three options…So, maybe I'll go to the list here. Because I don't really, yeah, and I'm going to the list because the map, like that might take a few minutes to figure out. I know what a list looks like.' P14*
	iii. RecMap website functionality	Parents liked the search feature and filters but experienced some barriers.	*'I like that there's a search right here. It's easy to find. So, if I was just coming for something specific, I could just put it in there.' P8*<br><br>*'I wouldn't say [that] searching for what I wanted, I got the result that I wanted necessarily. Maybe as I scroll down more will come up, but as the first kind of few things it's not there.' P16*
		Parents liked other RecMap website features but experienced some barriers	*'I like that top bar. It's very handy for once you get familiar with the page. I could just go over there rather than scrolling up and down incessantly looking for things. That's really handy.' P26*<br><br>*'This also takes a long time [to load]… Load time is a big thing. People are extremely impatient now and everyone wants things as quick and fast and they know that if your website doesn't give it, they'll just go to the next and get it. So, I think load time is like one of the top priorities for a website.' P13*

**3) Perceived usefulness of the RecMap website**	i. Intentions to use the RecMap website	Parents have intentions to use the RecMap website in the future and share it with others	*' I definitely would use it and I'd recommend it to other people.' P25*<br>*'I mean I definitely would recommend if someone was specifically looking for information and they wanted to know the most up to date and reputable stuff, this certainly makes sense. Because when you do just use Google, you do certainly have to look through a bunch of information that just – you wouldn't want to use.' P12*
		Parents would discuss the RecMap website with their healthcare provider or seek out support information	*'…like if they didn't understand something, they could print it off from your website and then they could take it to their doctor and say, I don't understand this. Can you please explain this to me?...' P25*<br>*'I'll read it. Then I'll probably verify it with something [that] is more reliable.' P21*
	ii. Awareness and expectations of the RecMap website	Parents had limited prior knowledge and awareness about the RecMap website	*'No [I have not heard of this website], not prior to this study…I'm not sure [why], to be honest. Maybe it just hasn't been referred from a person that I follow or an affiliate link from some other government site, or just based on the ones that I follow, it hasn't kind of fallen into my path, I would say.' P13*
		Parents liked the overall concept of the RecMap website	*'…it's almost like a COVID-19 [search engine], which is nice. It's like you can enter anything and then hopefully you find your answers. So, that is definitely good…I mean I think the idea of the website is great. Like the fact that you can come here and get information that is from reputable sources. You can search things, there's definitely different ways that you can find [information]. I think it is a great website to use.' P12*
		Some components of the RecMap website were not what parents expected	*'I was expecting pictures of families and bigger titles that are pretty clear and if you're a parent and you are looking for information, you do want to trust its source, but you also want it to be clear cut. You don't have time to go digging if there's a kid puking next to you. You want like should I go to the hospital right now? What am I supposed to watch for if my kid is sick? You probably haven't slept last night.' P9*
**4) Knowledge mobilization strategies**	i. Recommendations to tailor the RecMap website for parents	Parents would like to see the RecMap website tailored to parent preferences	*'Yes, that's what I would recommend…two different portals [split for lay and academic audiences] which I think it would be beneficial because this is excellent information for people that are not parents as well. People that work with children, work with parents, work with communities, populations, etc., … I think there's just a different way of trying to not meet everybody's needs and have separate portals.' P2*<br>*'I think putting plain language as a priority of information and having the option of the scientific language secondary would be the biggest win.' P13*

		Parents felt that RecMap website accessibility needs to be improved	*'From accessibility point of view, I think it is more recommended to have a contrast of colour which is mentioned in the accessibility guidelines… an example that this white and black is more readable as compared to this light green and white.' P6*
	ii. Approaches to dissemination	Parent would like to see information about the RecMap website shared in convenient and high traffic locations where parents frequent often	*'…you can have reputable individuals sharing the website forward. I think that's a useful way or even having advertisements or notices of it in places that parents would be accessing. So, I mean we talked about shared platforms, but maybe in the schools, like a school newsletter or on the bulletin board at the school or at the doctor's office. Those kinds of places that parents would be going.' P16*<br>*'…places where parents are more likely to read things. I mean, old school, but kid's magazines, kids sporting events, etc., to reach a demographic of parents.' P1*
		Parents would like to see the RecMap website share in places that are reputable and trusted	*'I would probably want like a trusted source to mention it to me. Word of mouth probably the best way [and] even just like describing it as a way of like the ultimate resource of COVID information for parents or something.' P9*
		Parents would like the information about the RecMap website to be shared in a variety of formats and be captivating	*'I've come to understand that you would need to do all three [link, QR code, and handout]. Some parents are able to use technology, and I'm quite familiar with QR codes. But I think that would be very overwhelming to some parents. And especially if you have English language as not a first language for parents. I think a paper pamphlet recommendation might be able to be more helpful there as well. I would suggest different parents have different means to accessing information. And you'd probably be reaching a large demographic of parents by doing more than one.' P1*<br>*'For example, you have added a new guideline in your website and you want people to know about it then instead of just having a story [on social media] – have a story about the website, have a story about the recommendation and then people can click for more information and go to the website…That will engage more people as opposed to just advertising the website.' P11*

COVID-19, Coronavirus Disease 2019; RecMap, Recommendations Map & Gateway to Contextualization.
