# Supplementary material for: Parents’ User Experience Accessing and Using a Web-Based Map of COVID-19 Recommendations for Health Decision-Making: Qualitative Descriptive Study
Source: JMIR Form Res. 2024 Mar 20;8:e53593. doi: 10.2196/53593 (PMC10956570; doi:10.2196/53593)
Supplement: Multimedia Appendix 3 [file formative_v8i1e53593_app3.pdf]

S1

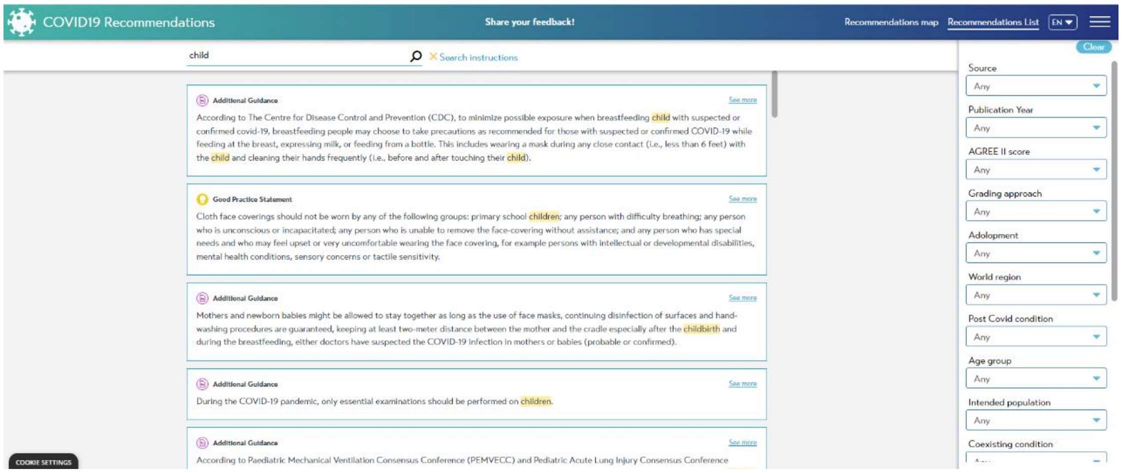

S2)

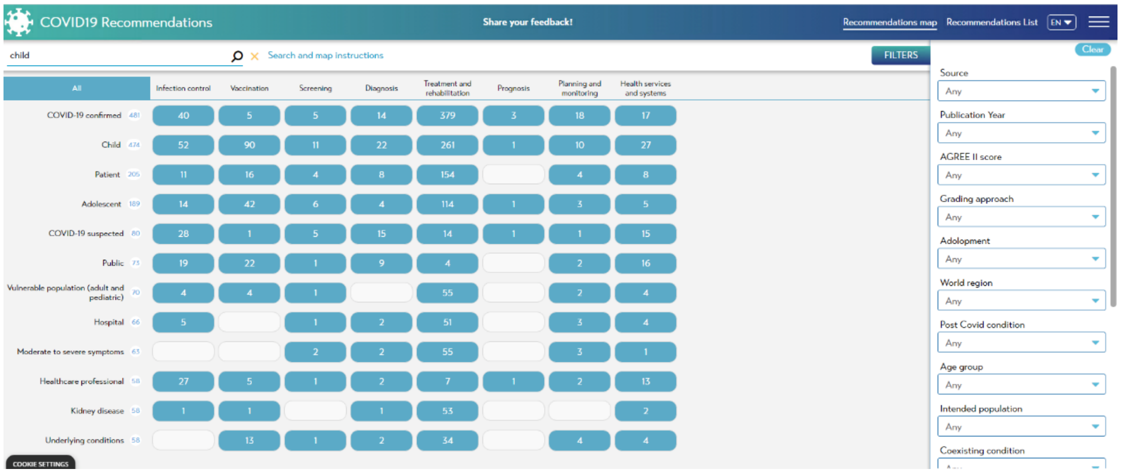

Screenshots are from the RecMap website (https://covid19.recmap.og), October 2023 [3].
